# pH mediated assemblage of carbon, nitrogen, and sulfur related microbial communities in petroleum reservoirs

**DOI:** 10.3389/fmicb.2022.952285

**Published:** 2022-09-14

**Authors:** Yang Li, Yuanyuan Zhang, Sheng Xue

**Affiliations:** ^1^State Key Laboratory of Mining Response and Disaster Prevention and Control in Deep Coal Mines, Anhui University of Science and Technology, Huainan, China; ^2^School of Safety Science and Engineering, Anhui University of Science and Technology, Huainan, China; ^3^Joint National-Local Engineering Research Centre for Safe and Precise Coal Mining, Anhui University of Science and Technology, Huainan, China

**Keywords:** petroleum acidification, functional communities, degradation, co-occurrence network, keystone taxa

## Abstract

Microorganisms are the core drivers of biogeochemistry processes in petroleum reservoirs and have been widely used to enhance petroleum recovery. However, systematic information about the microbial communities related to the C-N-S cycle in petroleum reservoirs under different pH conditions remains poorly understood. In this study, 16S rRNA gene data from 133 petroleum samples were collected, and 756 C-N-S related genera were detected. The Chao1 richness and Shannon diversity indices for the C-N-S-related microbial communities showed significant differences among different pH conditions and at the lowest levels in acidic conditions with pH values of 4.5–6.5. In addition, pH was the most important factor influencing the C-N-S related microbial communities and contributed to 17.95% of the variation in the methanogenesis community. A total of 55 functional genera were influenced by pH, which accounted for 42.08% of the C-N-S related genera. Among them, the genera *Pseudomonas* and *Arcobacter* were the highest and were concentrated in acidic conditions with pH values of 4.5–6.5. In parallel, 56 predicted C-N-S related genes were examined, and pH affected 16 of these genes, including putative chitinase, *mcrA, mtrB, cysH, narGHIVYZ, nirK, nirB, nifA, sat, aprAB*, and *dsrAB*. Furthermore, the co-occurrence networks of the C-N-S related microbial communities distinctly varied among the different pH conditions. The acidic environment exhibited the lowest complex network with the lowest keystone taxa number, and *Escherichia-Shigella* was the only keystone group that existed in all three networks. In summary, this study strengthened our knowledge regarding the C-N-S related microbial communities in petroleum reservoirs under different pH conditions, which is of great significance for understanding the microbial ecology and geochemical cycle of petroleum reservoirs.

## Introduction

Microorganisms are the core drivers of the biogeochemistry of important elements (especially C, N, and S) in petroleum reservoirs and have received extensive attention for their ability to enhance the recovery of energy materials such as petroleum (Brown, 2010; Nazina et al., 2019; Zhou et al., 2020). The distribution characteristics of microbial populations in global petroleum reservoirs have been extensively reported in the past decade (Aurepatipan et al., 2018; Zhou et al., 2019, 2020; de Sousa Pires et al., 2021). In addition, a variety of C-N-S related functional microorganisms, including sulfate reducers, fermentative bacteria, acidogens, and methanogens (Magot et al., 2000; Li et al., 2017b; Santos et al., 2020), have been widely detected in petroleum reservoirs. All of these functional taxa are inseparable from the exploitation and development of petroleum reservoirs. However, there is still a lack of a systematic description of these groups in petroleum reservoirs, which is crucial for understanding the C, N and S cycles in petroleum reservoirs.

Petroleum reservoirs are often considered extreme environments because of their physico-chemical properties, such as extremely variable pH and salinity, and are almost completely anaerobic (Varjani and Gnansounou, 2017). Under petroleum reservoir anaerobic environments, the coexistence and/or competition of different functional microorganisms could directly affect the quality of petroleum and the efficiency of petroleum recovery. In particular, the current microbial enhanced oil recovery (MEOR) technology is widely used to extract residual petroleum from low-productivity reservoirs by converting hydrocarbons through microbial fermentation and methanogenesis (Meslé et al., 2013; Quraishi et al., 2021). Previous studies have found that petroleum hydrocarbons can be degraded by a variety of microorganisms following a quasi-step-by-step biodegradation process (Meslé et al., 2013). Petroleum hydrocarbons are first degraded into single molecules and oligomers by petroleum hydrocarbon-degrading bacteria and fermentative bacteria, and then, some intermediate products are generated by different acidifying bacteria, acetic acid-producing bacteria and hydrogen-producing bacteria. The products from the degradation and fermentation of petroleum hydrocarbons could provide basic growth substrates for the hydrogenotrophic and acetate methanogenesis archaea (Magot et al., 2000). In addition, homotrophic oxidation pathways coupled to hydrogenotrophic methanogenesis are prevalent in many cases (Liang et al., 2015; Li et al., 2016). Because of the synergistic cooperation of C-N-S-related communities, including methanogens, petroleum hydrocarbon-degrading bacteria and fermentative bacteria, the petroleum degradation process has thermodynamic advantages under anaerobic conditions (Mbadinga et al., 2011). However, pH is an important factor that leads to changes in these functional groups (Zagrodnik and Łaniecki, 2017; Li et al., 2021).

The pH change in petroleum is closely related to sulfate-reducing bacteria (SRB). SRBs are of particular interest because they are associated with petroleum bioacidification and biocorrosion of metals used in the petroleum industry, which consequently result in huge financial losses (Varjani and Gnansounou, 2017; Narenkumar et al., 2019). Therefore, the implementation of MEOR technology often requires the addition of nitrate to stimulate the activity of nitrate-reducing bacteria (NRB) and inhibit the growth of SRB. It could be used to reduce sulfide generation caused by the sulfate reduction process, which could control the deterioration of petroleum quality and corrosion of engineering materials (Gao et al., 2013). Therefore, understanding petroleum C-N-S related microbes is beneficial to apply microbial knowledge to the actual production in the petroleum industry.

The co-occurrence relation of C-N-S-related functional groups is also important for community functional stability. For example, SRB in anaerobic environments are able to use sulfate as electron acceptors to provide biosulfur by the assimilation pathway for the production of amino acids and proteins (Mansilla et al., 2000; Tikariha and Purohit, 2019) and can also excrete H_2_S through the dissimilation pathway (Santos et al., 2020). Such dissimilation processes can still provide H^+^ for methanogens and are beneficial to support their carbon resource utilization (Muyzer and Stams, 2008; Tang et al., 2009). These mutual relationships among microorganisms, especially symbiosis, competition, predation, etc., can be reflected by the microbial co-occurrence network (Mougi and Kondoh, 2012; Qian and Akçay, 2020). Closely related microbes would allow them to occupy favorable ecological niches, and communities would become more stable against habitat change (i.e., pH changes). In general, keystone groups are often considered to have irreplaceable roles in microbial community structure and function (Ma Q. et al., 2020; Zhou et al., 2022) due to their unique topological properties, and they are also the core members of linking modules and networks (Coux et al., 2016; Xue et al., 2018). Keystone groups can be estimated based on the topological roles of nodes in networks, which are concatenated to microbial communities and provide new insights into the architecture in microbial co-occurrence networks (Banerjee et al., 2018; Wang et al., 2020). However, little is known about the co-occurrence networks of C-N-S-related functional groups in petroleum reservoirs and the keystone driver taxa under different pH conditions.

In this study, 16S rRNA data of petroleum samples from the NCBI database were extracted and used to reanalyze the C-N-S-related microbial composition. This study aims to (1) describe the C-N-S-related microbial communities and functional genes in petroleum reservoirs under different pH conditions and (2) explore the co-occurrence network of C-N-S-related microbial communities and keystone taxa in petroleum reservoirs.

## Materials and methods

### Datasets

Until February 2022, literature retrieval was conducted through the Web of Science database, and the published papers on “petroleum” and “microbial communities” were retrieved (Lenchi et al., 2013; Gao P. et al., 2015; Gao et al., 2016; Mand et al., 2016; Shelton et al., 2016; Li et al., 2017a; Aurepatipan et al., 2018; Nazina et al., 2019; Phetcharat et al., 2019; Zhou et al., 2019, 2020; Santos et al., 2020; de Sousa Pires et al., 2021). Samples of petroleum or produced water that are closely related to petroleum reservoirs were manually selected. The fastQ files according to the accession numbers of the 16S rRNA gene from petroleum samples were downloaded. These 16S rRNA gene data from 133 petroleum samples were collected for meta-analysis. The detailed sample information is shown in Supplementary Table 1. To analyze the changes in the C-N-S-related microbial communities under different pH conditions, the petroleum samples were divided into three groups based on their pH values, namely, an acidic group with pH 4.5–6.5 (4.5 ≤ pH ≤ 6.5) (*n* = 31), a neutral group with pH 6.5–7.5 (6.5 < pH < 7.5) (*n* = 57), and an alkaline group with pH 7.5–9.0 (7.5 ≤ pH < 9.0) (*n* = 45).

### Bioinformatics analysis

For microbial community (bacteria and archaea) analysis, the reads from 16S genes were merged, and the raw sequences were quality filtered using the QIIME pipeline. The chimeric sequences were identified by the “identify_chimeric_seqs.py” command and removed with the “filter_fasta.py” command according to the UCHIME algorithm. The selection and taxonomic assignment of operational taxonomic units (OTUs) were performed based on the SILVA reference data (version 128) at 97% similarity. Reads that did not align to the anticipated region of the reference alignment were removed as chimeras by the UCHIME algorithm. Reads that were classified as “chloroplast,” “mitochondria,” or “unassigned” were removed.

The predictive functional abundance was predicted by PICRUSt2 (Phylogenetic Investigation of Communities by Reconstruction of Unobserved States) with “picrust2_pipeline.py”^1^ (Douglas et al., 2020), which performed 4 key steps, including sequence placement, hidden-state prediction of genomes, metagenome prediction and pathway-level predictions. In addition, the additional output file Predicted Enzyme Commission (EC) number copy numbers were used to screen the C-N-S-related microbial genera. These C-N-S-related microbial genera included carbon degradation (petroleum degradation) genera, methanogenesis genera, CH_4_ oxidation genera, N_2_ fixation genera, ammoxidation genera, denitrification genera, dissimilatory nitrate reduction to ammonium (DNRA) genera and sulfur reduction genera.

### Data analysis

To avoid differences in amplified fragments among different samples, the analysis was performed at the genus level. The Shannon diversity and Chao1 richness for the C-N-S-related and each single C/N/S-related microbial communities were determined according to the relative abundance of genera. In addition, Bray-Curtis dissimilarities were calculated based on the relative abundance matrix of the C-N-S-related and each single C/N/S-related microbial genera in the Vegan package of R v 4.1.2. Non-metric multidimensional scaling (NMDS) was applied based on the Bray-Curtis dissimilarities by Vegan’s metaMDS function. Variation partitioning analysis (VPA) was used to determine the effects of chemical properties on the structure of the C-N-S-related microbial communities by Vegan’s varpart function.

Co-occurrence networks for the C-N-S-related microbial genera were constructed by the SparCC method with a significance of *P* < 0.05 and correlation coefficient | R| > 0.40 on the integrated Network Analysis Pipeline (iNAP)^2^ (Feng et al., 2022). For each pH condition, the genera detected in more than 10% of samples were included for Spearman correlation analysis. The network properties were assessed by the “Global Network Properties and Individual Nodes’ Centralit” module. The within-module connectivity (Zi) and among-module connectivity (Pi) values were calculated by the “Module separation and module hubs” module. Based on the Zi and Pi values, the functional genera in co-occurrence work were classified into four topological roles: module hubs (Zi ≥ 2.5, Pi < 0.62), network hubs (Zi ≥ 2.5, Pi ≥ 0.62), connectors (Zi < 2.5, Pi ≥ 0.62) and peripherals (Zi < 2.5, Pi < 0.62) (Olesen et al., 2007). Among them, module hubs, network hubs and connectors have been considered microbial keystone taxa (Banerjee et al., 2016).

## Results

### The diversity of C-N-S-related microbial communities

**FIGURE 1:**
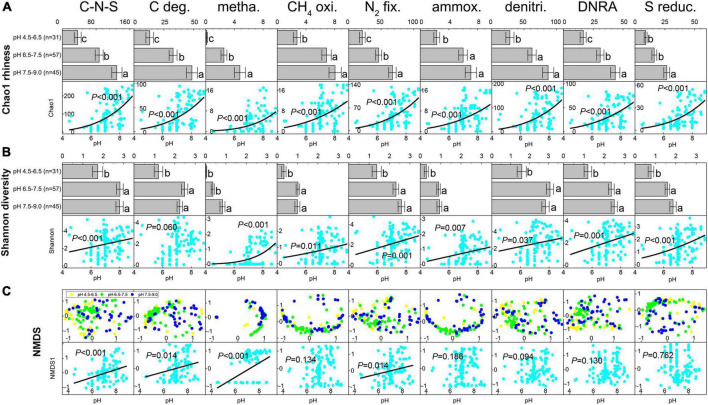
The shifts in Chao1 index **(A)** and Shannon index **(B)** for C-N-S-related microbial communities among different pH conditions. Difference letters indicated significant difference at *p* < 0.05. **(C)** Ordering of the composition of C-N-S-related microbial communities by non-metric multidimensional scaling (NMDS) using the Bray-Curtis distance index and the relationships between pH value and the first axes NMDS1 for the communities of C-N-S-related microbial communities. C-N-S, the total C-N-S-related microbial communities; C deg., carbon degradation; metha., methanogenesis; CH_4_ oxi., CH_4_ oxidation; N_2_ fix., N_2_ fixation; ammox., ammoxidation; denitri., denitrification; DNRA, dissimilatory nitrate reduction to ammonium; S reduc., sulfur reduction.

Based on the predicted EC number for each OTU in the petroleum microbial communities, a total of 756 C-N-S-related functional genera were detected, including 176 carbon degradation genera, 28 methanogenesis genera, 31 CH_4_ oxidation genera, 206 N_2_ fixation genera, 29 ammoxidation genera, 447 denitrification genera, 447 DNRA genera and 122 sulfur reduction genera (Supplementary Table 2). Among these 756 functional genera, 375 participated in multiple element cycles (Supplementary Table 2). For example, 26 known methanogens can fix N_2_. The Chao1 richness and Shannon diversity indices for the total C-N-S-related microbial communities ranged from 5 to 257 and 0.012 to 5.33, respectively, which showed lower numbers at pH 4.5–6.5 (Chao1 36.39 ± 8.21 and Shannon 1.73 ± 0.24, respectively, Figures 1A,B).

Among the relative abundance of the eight microbial groups, the relative abundance of N_2_ fixation and denitrification taxa was highest, followed by DNRA taxa, sulfur reduction taxa and carbon degradation taxa (Supplementary Table 3). The relative abundance of methanogenesis and ammoxidation taxa was the lowest. In addition, samples under acidic conditions with pH values of 4.5–6.5 exhibited the lowest relative number in methanogenesis but showed the highest relative abundance in CH_4_ oxidation, ammoxidation, denitrification, DNRA and sulfur reduction (Supplementary Table 3). In addition, the Chao1 richness and Shannon diversity indices among the eight microbial groups showed the lowest levels under acidic conditions with pH values of 4.5–6.5 (Figures 1A,B). The Chao1 richness and Shannon diversity indices in the majority of the eight microbial groups (except Shannon diversity in carbon degradation) increased with increasing pH (*P* < 0.05, Figures 1A,B).

**FIGURE 2:**
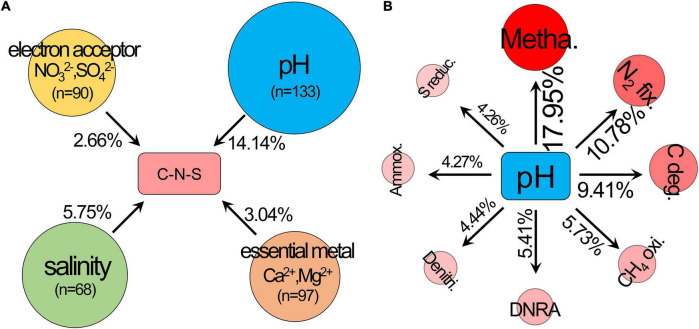
Variation partitioning analysis (VPA) was used to determine **(A)** the effects of pH, salinity, essential metal iron (Ca^2+^ and Mg^2+^) and electron acceptor (NO_3_^2–^ and SO_4_^2–^) on the structure of the C-N-S-related microbial communities, **(B)** the effects of pH on the microbial community structures of carbon degradation, methanogenesis, CH_4_ oxidation, N_2_ fixation, ammoxidation, denitrification, dissimilatory nitrate reduction to ammonium (DNRA) and sulfur reduction. C deg., carbon degradation; metha., methanogenesis; CH_4_ oxi., CH_4_ oxidation; N_2_ fix., N_2_ fixation; ammox., ammoxidation; denitri., denitrification; DNRA, dissimilatory nitrate reduction to ammonium; S reduc., sulfur reduction.

The ordering of samples by NMDS (Figure 1C) showed that the total C-N-S-related microbial structures among different pH values shifted by a small amount along the first axis (NMDS1). Herein, the first axes for the communities of C-N-S, methanogenesis, carbon degradation and N_2_ fixation were significantly correlated with pH (Figure 1C). VPA also showed that pH rather than salinity, the essential metal iron (Ca^2+^ and Mg^2+^) and electron acceptors (NO_3_^2–^ and SO_4_^2–^) were the most important factors influencing the C-N-S-related microbial communities (Figure 2A). The contribution of pH to the variation partitioning of the methanogenesis community was the highest (17.95%), followed by the communities of N_2_ fixation and carbon degradation (Figure 2B).

### The main C-N-S-related microbial genera and genes affected by pH

**FIGURE 3:**
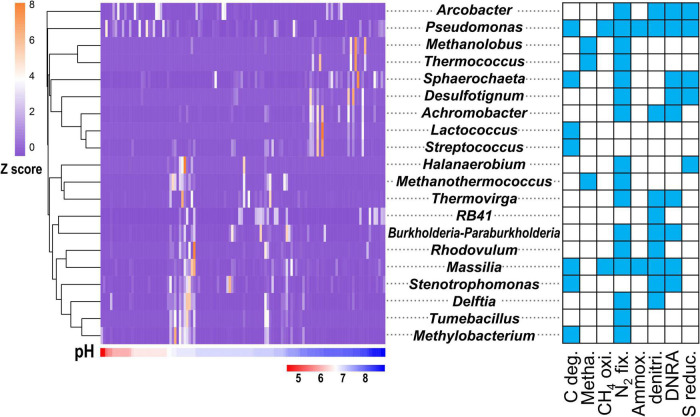
Relative abundance (exhibited by *Z* values) of top 20 genera affected by pH in the C-N-S-related microbial communities. The blue in the square indicates the corresponding function. The relative abundances of these genera are shown in Supplementary Table 4.

Among the 756 functional genera, 55 were influenced by pH (ANOVA, *P* < 0.05). These 55 functional genera (Supplementary Table 4) accounted for 30.20% of the total sequence reads and accounted for 42.08% of the C-N-S-related sequences. Among them, the top 20 genera (Figure 3) accounted for 29.53% of the total sequence reads, 18 of which had multiple functions. The genera *Pseudomonas* and *Arcobacter* were dominant and were mainly concentrated in acidic conditions with pH values of 4.5–6.5. The genus *Pseudomonas*, with most C-N-S functions except methanogenesis, showed the highest relative abundance (9.53 ± 1.72%) among the 55 functional genera, followed by *Arcobacter* (8.84 ± 2.12%). Some functional genera, including *Methanolobus, Thermococcus, Sphaerochaeta, Desulfotignum, Achromobacter, Lactococcus*, and *Streptococcus*, were concentrated in alkaline conditions with pH values of 7.5–9.0 (Figure 3). In addition, other genera were widely distributed in non-acidic environments with pH > 6.5. Among these genera, *Methanothermococcus, Methanolobus* and *Thermococcus* were dominant methanogenic groups and accounted for 5.22 ± 1.45%, 1.19 ± 0.55%, and 0.38 ± 0.13%, respectively.

**FIGURE 4:**
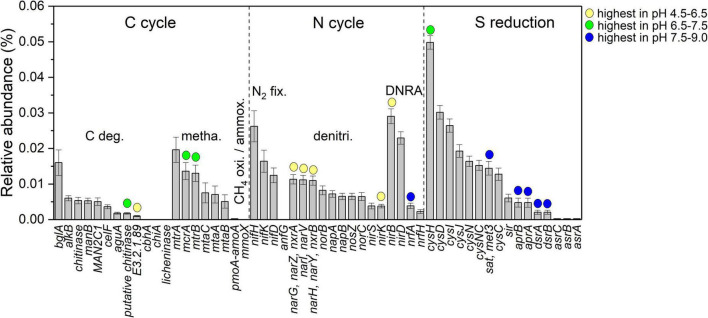
Comparison of different functional gene relative abundance among different pH conditions; yellow, green and blue circles indicate that the genes have the highest relative abundance in acidic condition with pH at 4.5–6.5, neutral condition with pH at 6.5–7.5 and alkaline condition with pH at 7.5–9.0, respectively (ANOVA with Tukey test, *P* < 0.05).

The relative abundance of 56 predicted C-N-S-related functional genes is shown in Figure 4. Among them, *the cysH* gene was the most abundant (4.98 × 10^–4^ ± 0.19 × 10^–4^). In addition, 16 genes were influenced by pH. Herein, putative chitinase (K03791, carbon degradation), *mcrA* (K00399, methanogenesis) and *mtrB* (K04480, methanogenesis) and *cysH* (K00390, sulfur reduction) genes in the neutral condition with pH at 6.5–7.5 were the highest in relative abundance among the samples. Genes E3.2.1.89 (K01224, carbon degradation), *narGHIVYZ* (K00370, K00371, K00374, denitrification), *nirK* (K00368, denitrification) and *nirB* (K00362, DNRA) in the acidic condition with pH at 4.5–6.5 were the highest in the relative abundances among the samples. The genes *nifA* (K03385, DNRA), *sat* (K00958, sulfur reduction), *aprAB* (K16950, K16951, sulfur reduction), and *dsrAB* (K11180, K11181, sulfur reduction) under alkaline conditions with pH values of 7.5–9.0 showed the highest relative abundance among the samples.

### Co-occurrence networks of the C-N-S-related microbial communities among different pH conditions

**FIGURE 5:**
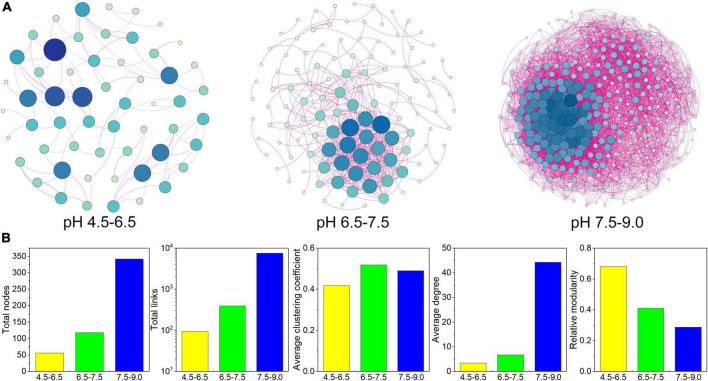
Co-occurrence network patterns of the C-N-S-related microbial communities among different pH conditions. **(A)** Visualization of constructed co-occurrence microbial networks. **(B)** Topological properties of the C-N-S-related microbial co-occurrence networks among different pH conditions.

The C-N-S-related co-occurrence networks among different pH conditions were constructed (Figure 5A) based on the 756 C-N-S-related functional genera. These three co-occurrence networks possessed scale-free topological properties, with *R*^2^ values of power-law ranging from 0.881 to 0.912 (Supplementary Table 5), which exhibited the non-randomized network structures. The multiple topological properties of the three C-N-S-related co-occurrence networks showed that the co-occurrence patterns shifted greatly among different pH conditions. The number of nodes increased from 56 to 342 with increasing pH, and the total links increased from 95 to 7577 with increasing pH (Figure 5B and Supplementary Table 5). In addition, microbial co-occurrence networks were also constructed (Supplementary Figure 1A). The relative abundances of C-N-S-related nodes to the total nodes decreased from 58.45 to 38.75% with increasing pH, and the relative abundances of the C-N-S-related links to the total links decreased from 80.61 to 63.48% with increasing pH (Supplementary Figure 1B).

In addition, the average clustering coefficient, average degree and connectedness of the C-N-S-related co-occurrence networks were the lowest in the acidic condition with pH at 4.5–6.5 (Figure 5B). Networks could be separated into 6, 22, and 4 modules under different pH conditions (Supplementary Table 5). In these modules, the number of nodes in the large modules (with at least 10 nodes) was 48.21% in the acidic condition with pH 4.5–6.5 and 100.00% in the alkaline condition with pH 7.5–9.0. Overall, these properties suggested that the C-N-S-related microbial communities under acidic conditions with pH values of 4.5–6.5 showed the lowest complex network.

**FIGURE 6:**
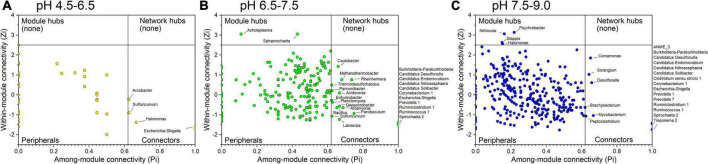
Identification of keystone taxa among different pH conditions based on their topological roles in networks. **(A)** acidic condition with pH at 4.5–6.5; **(B)** neutral condition with pH at 6.5–7.5; **(C)** alkaline condition with pH at 7.5–9.0. Module hubs are identified as Zi ≥ 2.5, Pi < 0.62, connectors are identified as Zi < 2.5, Pi ≥ 0.62, network hubs are identified as Zi ≥ 2.5, Pi ≥ 0.62.

A total of 0, 2, and 4 module hubs and a total of 4, 25, and 21 connectors were identified from the three networks in different pH conditions, respectively (Figure 6). Among them, *Escherichia-Shigella* was the connector that existed in all three networks. In addition, *Sulfuricurvum* was the connector detected in the networks of samples in acidic conditions with pH values of 4.5–6.5 and neutral conditions with pH values of 6.5–7.5. Several connectors between the networks of samples in the neutral condition with pH 6.5–7.5 and the alkaline condition with pH 7.5–9.0 were the same, including *Burkholderia-Paraburkholderia, Candidatus Desulforudis, Candidatus Endomicrobium, Candidatus Nitrososphaera, Candidatus Solibacter, Corynebacterium 1, Prevotella 1, Ruminiclostridium 1, Ruminococcus 1*, and *Spirochaeta 2*. All of these connectors, merely *Arcobacter* (connector in the acidic condition with pH at 4.5–6.5), *Burkholderia-Paraburkholderia and Spirochaeta 2*, were affected by pH (Supplementary Table 4).

## Discussion

This study predicted 8567 genes in petroleum microbial data, and the groups with these 56 C-N-S-related genes accounted for 70.38 ± 20.88% of the total microbial communities. Among them, nitrogen- and sulfur-related microbes accounted for a relatively high proportion of the total microbial communities in this study, indicating that most groups participated in the nitrogen and sulfur biogeochemical cycles of petroleum. These results demonstrated the important role of these petroleum microorganisms in the petroleum C-N-S cycle, and the stable microecology in petroleum reservoirs was inseparable from the synergy of multiple functional microorganisms. In addition, these groups are mainly related to biodegradation, methanogenesis, and sulfate reduction (Zhou et al., 2019; Santos et al., 2020; de Sousa Pires et al., 2021), which are key hubs to realize practical production.

The main factor influencing the C-N-S-related microbial community was pH in the reported petroleum samples. The microbial diversity at the genus level changed greatly among different pH conditions and showed the trend that the microbial diversity indices increased with increasing pH (Figure 1). The impact of pH on microorganisms widely exists in various anaerobic environments, including lake sedimentary environments (Banda et al., 2021) and wetlands (Kang et al., 2021). The petroleum reservoirs are anaerobic, and pH can change the anaerobic fermentation process, including anaerobic degradation of organic carbon and methanogenesis (Zhou et al., 2021; Lv et al., 2022). In addition, pH might have a greater effect on microbial groups related to methanogenesis, carbon degradation and N_2_ fixation. Methanogenesis is an important anaerobic respiration process in anaerobic environments, but the methanogenesis community had the lowest abundance and biodiversity among the C-N-S-related microbial communities (Figure 1 and Supplementary Table 2). These phylotypes had lower abundance and narrower tolerance to environmental conditions among these eight functional groups. Carbon degradation and N_2_ fixation can provide available carbon and nitrogen for petroleum microorganisms. In particular, most of the pH-regulated genera were closely related to carbon degradation and nitrogen fixation (Figure 3). Microbial growth is influenced by many factors, the most important of which is the availability of nutrients. Therefore, the availability of nutrients and competition for them determines microbial community assembly (Dubinkina et al., 2019). Although petroleum is mainly composed of carbon elements, the lack of available nutrients could also (especially available C and N) limit microbial activity (Ali Khan et al., 2017). In addition, nutrient stimulation has been considered to improve microbial activities and further stimulate oil emulsification and gas production (Gao et al., 2014). Therefore, the response of these groups to pH is mainly to control energy metabolism and nutrient metabolism in petroleum microhabitats, thereby maintaining the stability of microorganisms.

The main anaerobic respiration modes possessed differences under different pH conditions, such as sulfate reduction under alkaline conditions with pH 7.5–9.0, methanogenesis under neutral conditions with pH 6.5–7.5, and denitrification under acidic conditions with pH 4.5–6.5. This result indicated the difference in the main electron transport modes in petroleum microhabitats (Wartell et al., 2021), and as a result, electron acceptors were also important factors in regulating petroleum microorganisms (Figure 2). In the field of petroleum microorganisms, sulfate reduction is also a biochemical process that has attracted much attention. For example, SRB containing *dsr* genes are capable of sulfate reduction with iron as an electron donor, resulting in electrical microbially influenced corrosion (EMIC) (Santos et al., 2020). In addition, these microorganisms were capable of producing H_2_S through sulfate dissimilatory reduction. The accumulation of H_2_S can lead to acidification of the oil, which can damage the characteristics and quality of the oil, thereby posing an explosion threat and a threat to human health (Varjani and Gnansounou, 2017). This study found that the sulfur reduction genes here were mainly dominated by the *cys* genes, not the *dsr* genes encoding sulfate dissimilatory reduction (Figure 4), indicating that sulfate in petroleum reservoirs was mainly utilized by microbial communities through the assimilatory sulfate reduction pathway. Several Cys enzymes were used to synthesize sulfites and convert sulfates into sulfides, and the existence of sulfate utilization enhanced the bacterial ability to produce amino acids, such as cysteine and methionine (Tikariha and Purohit, 2019). This process provided biosulfur for the microbial communities in petroleum reservoirs.

This study showed C-N-S-related microbial co-occurrence networks in petroleum reservoirs with various patterns among different pH conditions, which were mainly determined by microbial keystone groups that survived under different pH conditions. The unique biological interactions of keystone taxa maintain the stability of functional microbial networks (Coux et al., 2016). Many studies have generally focused on the predominant microbial populations, while specific microorganisms, such as keystone taxa, could also play unique and important roles in community structure and function (Ma B. et al., 2020; Zhou et al., 2022). For example, *Pseudomonas* affected by pH showed a higher relative abundance and was dominant and mainly concentrated in acidic environments but did not exhibit keystone taxa in the microbial co-occurrence network. *Pseudomonas* is a bacterial genus that has been reported to be ubiquitous in microbial communities of petroleum reservoirs (Gao P. K. et al., 2015; Zhou et al., 2020). It is precisely because of the existence of multiple functions of this group that it has different metabolic potentials, allowing it to persist and grow in a wide range of petroleum reserve environments and to utilize a variety of carbon compounds under special environmental conditions. Their lifestyle may be opportunotrophic, as described by Singer et al. (2011). In addition, Vick et al. (2019) observed two *Pseudomonas* species with markedly different metabolic and ecological lifestyles, reflecting the broad metabolic and lifestyle diversity within such taxa, from parasitic to mutually beneficial (Polz et al., 2006) and free-living lifestyles. Among the keystone taxa, only *Escherichia-Shigella* was the key connector that existed in all three networks, which showed that the keystone taxa maintaining the C-N-S microbial network changed dramatically with shifting pH. *Escherichia-Shigella* did not change in relative abundance under different pH conditions, which has not received widespread attention in the field of petroleum microorganisms, but it is considered to play an important role in the degradation of petroleum hydrocarbons (Cui et al., 2020). In addition, most of the keystone taxa were the same in non-acidic environments (pH > 6.5) but were different from those in acidic conditions. It was suggested that acidity leads to the reduction of keystone taxa and may eventually lead to the collapse of modules and networks (Coux et al., 2016; Liu et al., 2022). The genus *Sulfuricurvum* coexists in neutral and acidic conditions (Figure 6). It may be an important keystone taxon involved in nitrogen and sulfur metabolism in petroleum reservoirs. They grow best in pH 7.0 and low-intensity salt medium and use sulfide, elemental sulfur, thiosulfate and hydrogen as electron donors and nitrate as an electron acceptor under anaerobic conditions (Kodama and Watanabe, 2004).

## Conclusion

This study comprehensively demonstrated the C-N-S-related microbial communities and functional genes in petroleum reservoirs under different pH conditions. Nitrogen- and sulfur-related microbes accounted for a relatively high proportion of the total microbial communities in petroleum reservoirs. In addition, the pH was the main factor influencing the C-N-S-related microbial diversities and microbial groups participating in methanogenesis, carbon degradation and N2 fixation. In addition, C-N-S-related microbial co-occurrence networks in petroleum reservoirs showed various patterns with different microbial keystone groups among different pH conditions. The interrelationship of these C-N-S-related microbial communities ultimately affects the microhabitat in petroleum reservoirs and has important implications for the decomposition of organic matter in petroleum reservoirs and the geochemical cycle.

## Data availability statement

The original contributions presented in this study are included in the article/Supplementary material, further inquiries can be directed to the corresponding author/s.

## Author contributions

YL, YZ, and SX conducted the bulk of the data analysis for the study and co-wrote the manuscript. YL and SX provided the funding for the study and were involved in the conceptualization of the study, as well as assisting in writing of the manuscript. All authors read and approved the final manuscript.

## Footnotes

1
https://github.com/picrust/picrust2

2
http://mem.rcees.ac.cn:8081/
