# Supplementary material for: pH mediated assemblage of carbon, nitrogen, and sulfur related microbial communities in petroleum reservoirs
Source: Front Microbiol. 2022 Sep 14;13:952285. doi: 10.3389/fmicb.2022.952285 (PMC9515653; doi:10.3389/fmicb.2022.952285)
Supplement: Supplementary file 1 [file Data_Sheet_1.DOCX]

**Title Page**

**1. Type of contribution**

Research paper

**2. Title**

pH-mediated the assemblage of carbon, nitrogen and sulfur related microbial communities in petroleum reservoirs

**3. Author names**

Yang Li^1,*^, Yuanyuan Zhang^2^, Sheng Xue^2,3^

**4. Author affiliations**

^1^ State Key Laboratory of Mining Response and Disaster Prevention and Control in Deep Coal Mines, Anhui University of Science and Technology, Huainan, Anhui province, China

^2^ School of Safety Science and Engineering, Anhui University of Science and Technology, Huainan, Anhui province, China

^3^ Joint National-Local Engineering Research Centre for Safe and Precise Coal Mining, Anhui University of Science and Technology, Huainan, Anhui province, China

**5. Corresponding author^*^**

Dr. Yang Li

Email: [liyang20130104@163.com; liyang_aust@163.com](mailto:liyang20130104@163.com;%20liyang_aust@163.com)

ORCID: 0000-0002-8946-3962

**6. Present address**

No. 168, Taifeng Road, Anhui University of Science and Technology, Huainan, Anhui province, China, 232001

**7. Manuscript information**

Number of figures: 6

Number of supplementary materials: 5 tables and 1 figure


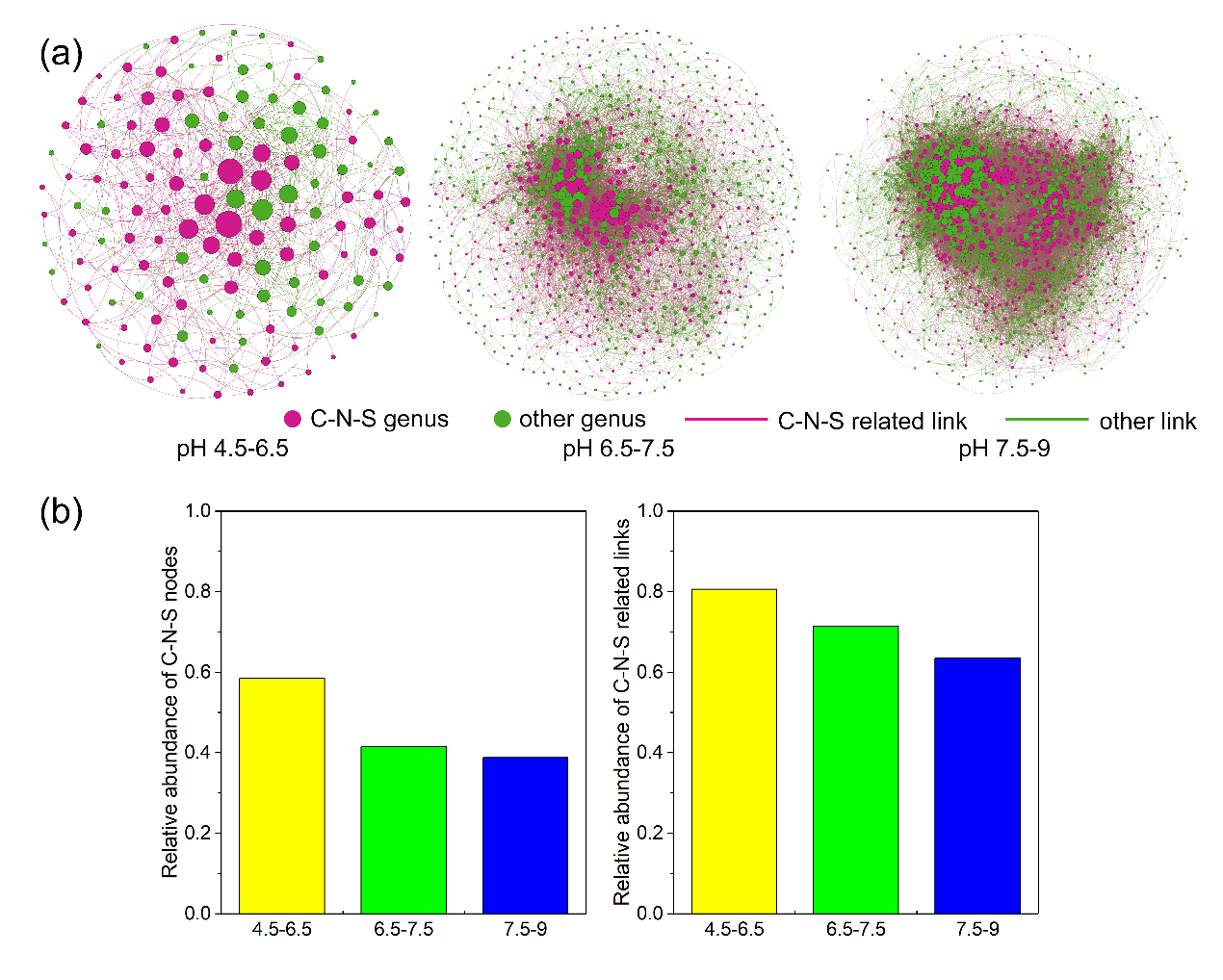


Fig. S1 Co-occurrence network patterns of microbial communities among different pH conditions. (a) Visualization of constructed co-occurrence microbial networks with C-N-S related nodes and links. (b) Topological properties of the microbial co-occurrence networks with C-N-S related nodes and links among different pH conditions. Other genus and other link mean that the genus and the link were not related to C-N-S function.

Table S1 The detailed sample information of microbial communities in petroleum samples

Table S2 List for C-N-S related functional genera in petroleum samples

Table S3 Relative abundance (%) of the total functional genera in petroleum samples. Different capital letters indicate differences of the total relative abundance among functional genera (*P* < 0.05). Different lowercase letters indicate differences in the total relative abundance of functional genera among different pH (*P* < 0.05).

|  | C degradation | Methanogenesis | CH_4_ oxidation | N_2_ fixation | Ammoxidation | Denitrification | DNRA | S reduction |
| --- | --- | --- | --- | --- | --- | --- | --- | --- |
| total | 27.66 ± 2.49C | 10.66 ± 1.96D | 11.80 ± 1.77D | 54.61 ± 2.50A | 11.23 ± 1.76D | 50.92 ± 2.85A | 40.26 ± 2.88B | 33.32 ± 2.80BC |
| 4.5-6.5 | 30.57 ± 6.14a | 0.00 ± 0.00b | 21.49 ± 5.74a | 61.74 ± 6.01a | 21.42 ± 5.73a | 68.59 ± 6.08a | 59.94 ± 6.21a | 56.19 ± 6.38a |
| 6.5-7.5 | 23.97 ± 2.98a | 17.18 ± 3.53ab | 7.77 ± 1.29b | 55.45 ± 3.65a | 6.53 ± 1.12b | 44.77 ± 3.94b | 35.70 ± 4.13b | 26.17 ± 3.95b |
| 7.5-9.0 | 30.27 ± 4.72a | 9.83 ± 3.26a | 10.18 ± 2.71b | 48.69 ± 3.89a | 10.12 ± 2.71b | 46.50 ± 4.77b | 32.49 ± 4.39b | 26.59 ± 3.65b |

Table S4 The relative abundances (%) of the C-N-S related functional genera those affected by pH

Table S5 Topological properties of the co-occurrence networks for C-N-S related microbial communities among different pH.

| Network indexes | pH 4.5-6.5 | pH 6.5-7.5 | pH 7.5-9.0 |
| --- | --- | --- | --- |
| Similarity threshold | 0.4 | 0.4 | 0.4 |
| Total nodes | 56 | 118 | 342 |
| Total links | 95 | 397 | 7577 |
| R square of power-law | 0.881 | 0.912 | 0.907 |
| Average degree | 3.393 | 6.729 | 44.31 |
| Average clustering coefficient | 0.419 | 0.519 | 0.491 |
| Average path distance | 4.119 | 3.353 | 2.187 |
| Connectedness | 0.314 | 0.494 | 0.466 |
| Efficiency | 0.988 | 0.991 | 0.981 |
| Modularity | 0.681 | 0.411 | 0.288 |
| No. of modules | 6 | 22 | 4 |
| No. of large modules* | 2 | 3 | 4 |
| No. of nodes in large modules* | 27 | 59 | 342 |
| Percentages of nodes in large modules* (%) | 48.21 | 50.00 | 100.00 |

* Large modules contain at least 10 nodes.
